# Functional Classification of *TP53* Mutations in Acute Myeloid Leukemia

**DOI:** 10.3390/cancers12030637

**Published:** 2020-03-10

**Authors:** Sayantanee Dutta, Gudrun Pregartner, Frank G. Rücker, Ellen Heitzer, Armin Zebisch, Lars Bullinger, Andrea Berghold, Konstanze Döhner, Heinz Sill

**Affiliations:** 1 Division of Hematology, Medical University of Graz, A-8036 Graz, Austria; sayantanee.dutta@medunigraz.at (S.D.); armin.zebisch@medunigraz.at (A.Z.); 2 Institute for Medical Informatics, Statistics and Documentation, Medical University of Graz, A-8036 Graz, Austria; gudrun.pregartner@medunigraz.at (G.P.); andrea.berghold@medunigraz.at (A.B.); 3 Department of Internal Medicine III, University Hospital of Ulm, D-89081 Ulm, Germany; Frank.Ruecker@uniklinik-ulm.de (F.G.R.); konstanze.doehner@uniklinik-ulm.de (K.D.); 4 Institute of Human Genetics, Diagnostic and Research Center for Molecular Biomedicine, Medical University of Graz, A-8010 Graz, Austria; ellen.heitzer@medunigraz.at; 5 Otto-Loewi-Research Center for Vascular Biology, Immunology and Inflammation, Division of Pharmacology, Medical University of Graz, A-8010 Graz, Austria; 6 Department of Hematology, Oncology and Tumor Immunology, Charité University Medicine, D-10117 Berlin, Germany; lars.bullinger@charite.de

**Keywords:** acute myeloid leukemia, *TP53* mutations, functional classification, relative fitness core, prognosis

## Abstract

Mutations of the *TP53* gene occur in a subset of patients with acute myeloid leukemia (AML) and confer an exceedingly adverse prognosis. However, whether different types of *TP53* mutations exert a uniformly poor outcome has not been investigated yet. Here, we addressed this issue by analyzing data of 1537 patients intensively treated within protocols of the German-Austrian AML study group. We classified *TP53* mutations depending on their impact on protein structure and according to the evolutionary action (EAp53) score and the relative fitness score (RFS). In 98/1537 (6.4%) patients, 108 *TP53* mutations were detected. While the discrimination depending on the protein structure and the EAp53 score did not show a survival difference, patients with low-risk and high-risk AML-specific RFS showed a different overall survival (OS; median, 12.9 versus 5.5 months, *p* = 0.017) and event-free survival (EFS; median, 7.3 versus 5.2 months, *p* = 0.054). In multivariable analyses adjusting for age, gender, white blood cell count, cytogenetic risk, type of AML, and TP53 variant allele frequency, these differences were statistically significant for both OS (HR, 2.14; 95% CI, 1.15–4.0; *p* = 0.017) and EFS (HR, 1.97; 95% CI, 1.06–3.69; *p* = 0.033). We conclude that the AML-specific RFS is of prognostic value in patients with TP53-mutated AML and a useful tool for therapeutic decision-making.

## 1. Introduction

Acute myeloid leukemia (AML) is a heterogeneous aggressive malignancy occurring de novo, secondary to antecedent hematological disorders, or following cytotoxic treatments for a primary disease [1,2,3,4]. It is the most common acute leukemia in adults with an annual age-adjusted incidence rate of 3.5/100,000 men and women rising to 15–20/100,000 above the age of 60 years [5]. Extensive work over the last decade employing next generation sequencing technologies has decoded the AML genome. The pathogenesis of AML represents a multistep process involving mutagenesis, epigenetic dysregulation, and formation of copy number aberrations [6,7,8,9]. Thereby, initial genetic aberrations transform hematopoietic stem and progenitor cells (HSPCs) into preleukemic stem cells (preLSCs) that retain their capability to differentiate into normal blood cells [10,11,12]. While preLSCs do not generate leukemia in vivo, leukemic stem cells (LSCs) representing a distinct population with self-renewal capacity are capable to induce and perpetuate leukemia [13]. Importantly, in the vast majority of AMLs, multiple and diverse genetic subclones are observed [14].

Although novel targeted treatment approaches have been developed for patients with AML, their prognosis is still dismal with 5-year-survival rates of 40%–45% in patients below the age of 65 years and less than 20% in patients aged 65 years or older [7,15]. Relapsed disease as a consequence of diverse molecular aberrations affecting dormant LSCs is the main reason for this dismal outcome [16,17,18,19,20]. In AML, parameters influencing treatment decisions as well as outcome include both patient-specific characteristics and leukemia-specific aberrations. Among the former, patient´s age and comorbidities are important variables; among the latter, the type of leukemia, white blood cell count, and genetic aberrations significantly influence response to induction therapy, type of consolidation treatment, and survival [21,22,23]. An AML risk classification scheme based on cytogenetic abnormalities was proposed as early as in 1998 [24]. In the updated 2017 risk stratification of the European Leukemia Net (ELN), mutational aberrations play an increasingly important role [25]. Mutated *NPM1* as well as bi-allelic *CEBPA* mutations constitute favorable-risk parameters. Furthermore, the allelic burden of FLT3-internal tandem duplications was included as a prognostic parameter as assessed in a semi-quantitative manner. Importantly, mutations in the *TP53*, *RUNX1* and *ASXL1* genes, respectively, arose as novel, adverse risk factors.

Encoded by the tumor protein p53 (*TP53*) gene on chromosome 17p13.1, p53 is an essential cellular protein with response to cellular stress being one of its main functions [26,27]. It is expressed in HSPCs modulating quiescence and self-renewal thereby contributing to a constant lifelong pool of blood cells [28]. Aberrations of *TP53* are encountered in more than 50% of human malignancies. Thereby, the contribution of this gene towards tumorigenesis encompasses loss of wild type alleles and/or *TP53* mutations with certain mutant alleles exerting either dominant-negative or novel “gain-of-function” properties distinct from the null genotype [27,29,30,31]. Germline *TP53* mutations characterize the Li–Fraumeni and Li–Fraumeni-like syndromes conveying familial cancer predisposition with autosomal-dominant inheritance [32]. We described deleterious germline *TP53* mutations in patients with AML, preferably in therapy-related subtypes developing after ionizing irradiation [33,34]. In AML, somatically acquired *TP53* mutations constitute early events characterizing preLSCs [12,35]. They have been demonstrated to occur at a frequency of up to 10% in de novo cases, more than 20% in therapy-related myeloid leukemias, and up to 90% in erythroleukemias [7,36,37]. The majority of *TP53* aberrations represent missense mutations, in a number of cases accompanied by loss of the wild type allele. Most importantly, AML patients with *TP53* mutations show resistance to intensive treatment strategies, including allogeneic hematopoietic stem cell transplantation (HSCT) with 3-year overall survival rates between 0% and 15% [38,39,40]. Recently, a dismal outcome has also been described when *TP53* mutations occur in leukemic subclones with a variant allele frequency (VAF) of less than 20% [41].

However, it is largely unknown whether different types of *TP53* mutations—missense, nonsense, splice site mutations, as well as small insertions and deletions—exert a uniformly poor outcome in patients with AML. In this study, we investigated the prognostic impact of different *TP53* mutations using four *TP53*-specific functional scoring systems in a large cohort of intensively treated patients of the German-Austrian AML study group (AMLSG). We demonstrate that the AML-specific “Relative Fitness Score” (RFS) is capable of discriminating patients showing a significantly different overall survival (OS) and event-free survival (EFS), thereby serving as a novel tool for therapeutic decision-making.

## 2. Results

108 *TP53* mutations were detected in 98 of the 1537 patients investigated—88 (81.4%) missense, eight (7.4%) nonsense, and six (5.6%) splice site mutations, as well as six (5.6%) small insertions and deletions. In seven patients, we found two *TP53* mutations, and in one patient—four mutations (Table 1). According to the cytogenetic analysis, a sufficient number of metaphases was obtained in 84/98 (86%) patients showing a complex karyotype in 77 (92%) of them. The median VAF of the 108 *TP53* mutations detected in this cohort was 48.7% (range, 4.7% to 97%); concurrent gene mutations were reported previously [41]. The median follow-up of patients with wild type TP53 AML (*n* = 1439) was 895 days, and of patients showing *TP53* mutations—195 days, respectively.

**Figure 1:**
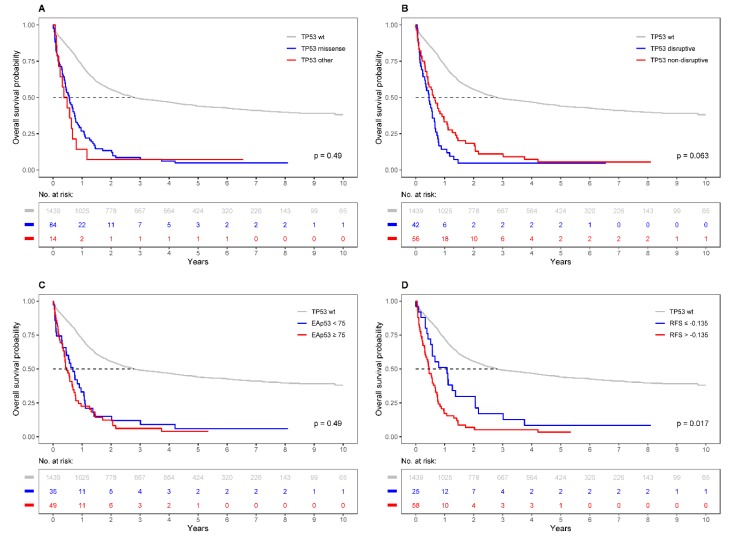
Overall survival (OS) probabilities for patients with *TP53*-mutated AML according to functional mutation scoring systems. (**A**) comparison of missense *TP53* mutations versus other types of aberration (nonsense and splice site mutations, insertions and deletions). (**B**) comparison of disruptive versus non-disruptive *TP53* mutations. (**C**) classification according to the “Evolutionary Action p53 Score”. (**D**) classification based on the “Relative Fitness Score” (RFS) with the AML-specific threshold of −0.135 showing a statistically significant survival difference (median OS, 12.9 versus 5.5 months). All *p*-values refer to the comparison of *TP53*-mutated groups. Abbreviation: wt, wild type.

Patients with a wild type *TP53* status showed a significantly better outcome than those with *TP53* mutations (median OS, 33.6 months versus 6.5 months; median EFS, 16.5 months versus 5.7 months) as described previously by our group and being in accordance with further reports [38,41,42]. The *TP53*-specific scoring systems applied in this study are described in detail in the “Materials and Methods” section. When comparing the impact of *TP53* missense mutations versus truncating mutations, we found 84 patients in the former group and 14 patients in the latter group, respectively. However, we did not find a significant difference with respect to OS and EFS using this scoring system. Next, we classified mutations into “disruptive” and “non-disruptive”, and identified 42 patients with at least one disruptive mutation and 56 with only non-disruptive mutations in the AMLSG cohort. Again, no significant difference in the outcome parameters analyzed was observed. We then investigated the EAp53 score focusing on patients with missense *TP53* mutations and found 49 patients in the high-risk and 35 patients in the low-risk group. Using this functional scoring system, we did not detect a significant survival difference between the two groups, either (Figure 1, Table 1 and Table 2, Figure S1, and Tables S1–S3).

The RFS was extracted for those 83 patients with *TP53* mutations located in the DNA-binding domain (DBD); 76 patients were classified as high-risk (RFS > −1), and seven patients—as low-risk (≤−1). Notably, patients with a low-risk RFS showed a significantly better OS (median, 13.4 versus 6.3 months; *p* = 0.019) and EFS (median, 11.7 versus 5.3 months; *p* = 0.024) (Table 1 and Table S1, Figure S2). These parameters remained borderline significant in multivariable analyses adjusting for age, gender, white blood cell count, cytogenetic risk, type of AML and *TP53* VAF, indicating a potential prognostic value of this score (Table 3). In a subsequent receiver operating characteristic (ROC) analysis, the optimal RFS cut-off value for AML patients was −0.135 (Figure S3) resulting in 25 low-risk and 58 high-risk patients, respectively. Using this leukemia-specific threshold, we found a significantly better OS (median, 12.9 versus 5.5 months, *p* = 0.017) and a trend towards improved EFS (median, 7.3 versus 5.2 months, *p* = 0.054) for patients with a low-risk RFS (Figure 1, Tables S2 and S3, Figure S1). In multivariable regression analyses including the established AML risk factors, the difference revealed statistically significance for both OS (HR, 2.14; 95% CI, 1.15–4.0; *p* = 0.017) and EFS (HR, 1.97; 95% CI, 1.06–3.69; *p* = 0.033) (Table 3).

## 3. Discussion

In patients with AML, *TP53* mutations confer an exceedingly adverse prognosis. Here, we applied four different *TP53*-specifc functional scoring systems to test whether particular mutations are associated with a better therapeutic outcome. Comparing missense *TP53* mutations with truncating aberrations did not show a difference with respect to OS and EFS of intensively treated AML patients. Our results, therefore, suggest that gain-of-function properties postulated for at least some of missense *TP53* mutations are not the predominant mechanism of therapeutic resistance in that cohort [43,44]. They are in accordance with recently published data indicating a dominant-negative effect of missense *TP53* mutations rather than acquisition of novel oncogenic properties in myeloid malignancies based on in vitro and in vivo analyses, as well as clinical data [45]. Discrimination of *TP53* mutations into disruptive and non-disruptive ones showed a statistically significant impact on survival in patients with head and neck cancers [46]. Furthermore, the EAp53 score was successfully used to predict outcome in patients with larynx cancer [47]. However, both *TP53* functional scoring systems did not show a survival difference in the AML patients investigated here. This points to the fact that the cellular context of *TP53* aberrations plays a pivotal role with respect to biology as well as therapeutic resistance [27].

Treatment of patients with *TP53*-mutated AML remains challenging. Non-intensive therapies with hypomethylating agents—azacitidine and decitabine—are frequently offered, especially to elderly patients [48,49]. These drugs show acceptable response rates and favorable toxicity profiles. However, they represent a non-curative treatment, and patients usually succumb to leukemic progression within months. Recently, experimental approaches targeting *TP53* mutations in myeloid disorders have reached the clinical stage of development. APR-246 is a small molecule designed to shift mutant p53 towards a wild type confirmation, thus inducing apoptosis of neoplastic cells [50,51,52]. Synergistic effects of this drug and azacitidine have been demonstrated in vitro using cells from patients with *TP53*-mutated AML and myelodysplastic syndromes (MDS) [53]. In a multiphase 1b/2 trial, APR-246 was given in combination with azacitidine to patients with *TP53*-mutated MDS and oligoblastic AML with preliminary results showing encouraging response rates of 85% [54]. Nevertheless, to date, intensive chemotherapy followed by allogeneic hematopoietic stem cell transplantation (HSCT) remains the only curative approach for AML and MDS patients with *TP53* aberrations [39,40]. Facing different therapeutic options, it is, therefore, of importance to develop tools supporting proper therapeutic decision-making in this cohort of patients. In a recently published article, clinical parameters—preferably performance status and achievement of a complete remission following induction therapy—have been shown to be of prognostic relevance in patients with *TP53*-mutated AML [55]. Analyzing a large cohort of patients treated with intensive chemotherapy and HSCT here, we were able to show that the AML-specific RFS enables discrimination of a low-risk group with a median OS of 12.9 months and a high-risk group with a median OS of only 5.5 months. We, therefore, conclude that in addition to clinical parameters, the AML-specific RFS may be a useful tool to identify those patients with *TP53* mutations who still benefit from intensive treatment approaches.

## 4. Materials and Methods

The study was approved by the ethics committee of the University of Ulm, Ulm, Germany (vote number 148/10) and registered under “ClinicalTrials.gov NCT00146120”. It was conducted according to the Declaration of Helsinki, all the patients gave written informed consent. A list of study investigators and centers is provided in the Supplementary Material.

The AMLSG cohort analyzed consisted of 1537 patients with newly diagnosed AML (de novo AML, *n* = 1408; secondary AML, *n* = 61; therapy-related AML, *n* = 68) who received intensive treatments, including allogeneic HSCT within three multicenter clinical trials. Treatment protocols, patient characteristics, and outcome data have been reported previously [56,57,58]. Briefly, in trial AML-HD98A, 627 patients aged 18 to 65 years received induction therapy with idarubicin, cytarabine, and etoposide (ICE). High-risk subjects were offered allogeneic HSCT, intermediate-risk subjects—either allogeneic HSCT from a suitable related donor or, alternatively, intensive chemotherapy, and low-risk subjects received intensive chemotherapy. Trial AMLSG 07-04 had a similar design to AML-HD98A and included 737 patients aged 18 to 61 years who were randomized to induction therapy with ICE or ICE/all-trans retinoic acid (ATRA). Trial AML-HD98B included 173 patients aged 58 to 84 years who were randomized to ICE or ICE/ATRA induction therapy with further treatment based on response.

Diagnostic bone marrow (BM) or peripheral blood (PB) specimens were collected at the University of Ulm, Ulm, Germany, at study entry and processed by the Ficoll density gradient centrifugation to enrich for mononuclear cells (BM, *n* = 579; PB, *n* = 800; unknown, *n* = 158). In this respect, it is important to emphasize that the mutational landscape is conserved in PB specimens of patients with AML and myelodysplastic syndromes at diagnosis and during treatment, respectively [59]. Genomic DNA was analyzed by a targeted sequencing approach focusing on 111 genes associated with myeloid neoplasms as described previously [7]. With respect to *TP53*, all coding exons and flanking exon-intron boundaries were sequenced. The median coverage for TP53 was 157×, and the lower limit of detection was set at 5% mutant allele reads. Sequencing results were deposited in the European Genome-Phenome Archive (www.ebi.ac.uk/ega, accession number EGAS0000100275).

To assess the functional impact of *TP53* mutations in AML, we investigated four different *TP53*-specific scoring systems. First, we compared the impact of missense *TP53* mutations versus all other types of mutations—nonsense and splice site mutations, as well as small insertions and deletions. This classification is based on the fact that novel gain-of-function properties may predominantly be associated with missense mutations [44,60]. Next, we analyzed whether the location of a particular *TP53* mutation and the amino acid alteration is of prognostic value in our AML cohort, as this had previously been demonstrated for patients with solid tumors [46]. Therefore, “disruptive mutations” were classified as DNA sequence alterations that introduce a STOP sequence resulting in disruption of p53 protein production or DNA sequence alterations that occur within the L2 or L3 binding domains (codons 163–195 or 236–251) and replace an amino acid from one polarity/charge category with an amino acid from another category (Table 4). “Non-disruptive mutations” were classified as any mutation occurring outside the L2 or L3 binding domain (except stop mutations) or mutations within the L2 or L3 binding domains that result in replacement of an amino acid with another one from the same polarity/charge category.

We then investigated the evolutionary action score (EAp53) that focuses on missense *TP53* mutations and was designed and validated in patients with head and neck cancers [47]. This algorithm takes evolutionary sensitivity to sequence variation and amino acid conservation into account and scores mutations from 0 to 100 with wild type *TP53* sequences having a score of 0. We extracted the EAp53 score of those AMLSG patients showing missense mutations from the respective server (http://mammoth.bcm.tmc.edu/EAp53) and used the threshold of 75 from the initial publication to discriminate between low-risk (<75) and high-risk groups (≥75). Finally, we assessed the relative fitness score (RFS) recently developed for TP53 mutations located within the DBD of the gene as another indicator of their functional impact [61]. In that work, a catalogue of 9833 unique DNA sequence variants was generated in human p53-null cells, and their selective growth was assessed in in vitro cultures. Thereby, RFS represents the logarithm (base 2) of the median of the relative enrichment or depletion of a particular TP53 variant assessed at three time points. A high RFS indicates preferential expansion in culture representing higher fitness of the variant, whereas a low RFS pinpoints preferential depletion. We extracted the RFS for the DBD *TP53* mutations of the AMLSG cohort using an online data resource (GSE115072) and classified patients into high-risk (RFS > −1) and low-risk (≤−1) groups. This threshold was based on data from individuals with Li–Fraumeni syndrome showing a statistically significant association between RFS and age at primary tumor detection [61].

### Statistical Analyses

For each *TP53* functional score, we assessed its impact on OS and EFS as defined by the European Leukemia Net [25]. Thereby, OS is measured from trial entry until the date of death from any cause or last follow-up, EFS—from trial entry until the date of refractory disease, relapse from complete remission, death, or last follow-up. Survival times were compared between different functional *TP53*-mutated groups using Kaplan–Meier curves and the log-rank test. Wild type *TP53* patients are shown in the plots as a reference but were not considered when assessing group differences. We estimated median survival rates along with their 95% confidence intervals (CIs) from these analyses. Furthermore, we performed Cox regression analyses determining hazard ratios (HRs) with 95% CIs for each of the *TP53* functional scores and assessed the influence of age, gender, white blood cell count, cytogenetic risk, type of AML (de novo, secondary, therapy-related), and *TP53* VAF on OS and EFS. In the eight patients with multiple *TP53* mutations, only that mutation with the severest predicted impact was used for each of the respective scoring systems as depicted in Table 1.

Since the threshold for the RFS was determined in a cohort of patients with different types of cancer, we performed a ROC analysis to determine the optimal threshold for patients with AML. Given that only 6/98 (6.1%) TP53-mutated patients survived the whole observation time, we considered 1-year mortality as the outcome. We determined the best threshold using the Youden’s index that weights sensitivity and specificity equally [62]. The EAp53 score was not considered for this analysis as the boxplots showed similar scores for survivors and non-survivors after one year as shown in Figure S4 and Table S4. All statistical analyses were conducted using R version 3.5.3.

## 5. Conclusions

In this study, four different *TP53*-specific scores were evaluated for their prognostic impact in patients with *TP53*-mutated AML. The AML-specific relative fitness score was capable of identifying patients with a significantly better prognosis. We conclude that—in addition to clinical parameters—this score should be incorporated into the therapeutic decision algorithm of patients with such a malignancy.

## Figures and Tables

**Table 1:** Characteristics of *TP53* mutations found in AMLSG patients and their assessment in different scoring systems. In the 8 patients with more than one *TP53* mutation (marked in grey), the mutation used for analysis is depicted in red. Abbreviations: non-disr., non-disruptive mutations; EAp53 Score, Evolutionary Action p53 Score; RFS, Relative Fitness Score; NA, not applicable; “other” refers to nonsense and splice site *TP53* mutations, as well as to small insertions and deletions. “Frequency” refers to the cohort analyzed.

Study	ID	cDNA Change	Amino Acid Change	Variant Allele Frequency	Frequency	Missense (1) Other (0)	Disruptive (1) Non-Disr. (0)	EAp53 Score	RFS
07-04	17	c.461G>A	p.G154D	12.5	1×	1	0	70.67	−2.09007995
07-04	30	c.773A>C	p.E258A	23.28	1×	1	0	93.29	−0.1473427
07-04	32	c.817C>T	p.R273C	13.74	4×	1	0	84.5	−0.12541853
07-04	41	c.524G>A	p.R175H	71.93	7×	1	0	78.5	−0.14494686
07-04	76	c.869G>A	p.R290H	45.69	1×	1	0	16.59	−1.5430162
07-04	110	c.658T>C	p.Y220H	62.66	1×	1	0	43.94	−0.68522627
07-04	129	c.488A>G	p.Y163C	58.93	2×	1	0	70	0.12544937
07-04	133	c.713G>A	p.C238Y	96.33	1×	1	0	92.66	0.2148364
07-04	139	c.427G>A	p.V143M	8.7	1×	1	0	51.7	−1.33071158
07-04	177	c.524G>A	p.R175H	89.01	7×	1	0	78.5	−0.14494686
07-04	197	c.743G>A	p.R248Q	88.02	7×	1	1	78.95	−0.03958725
07-04	198	c.659A>G	p.Y220C	30.77	5×	1	0	72.52	0.21906403
07-04	204	c.1024C>T	p.R342*	44.36	1×	0	1		
07-04	204	c.796G>C	p.G266R	46.25	1×	1		91.41	0.30570903
07-04	214	c.743G>A	p.R248Q	77.72	7×	1	1	78.95	−0.03958725
07-04	228	c.503A>C	p.H168P	94.44	1×	1	1	78.86	−0.31621125
07-04	241	c.994-1G>A	NA	87.94	1×	0	1		
07-04	246	c.799C>T	p.R267W	46.36	1×	1	0	89.04	−0.64560831
07-04	251	c.817C>T	p.R273C	70.95	4×	1	0	84.5	−0.12541853
07-04	302	c.824G>A	p.C275Y	86.52	1×	1	0	93.47	0.40550255
07-04	421	c.826G>C	p.A276P	5.78	1×	1		73.15	−0.16222675
07-04	421	c.743G>A	p.R248Q	12.84	7×	1	1	78.95	−0.03958725
07-04	421	c.587G>A	p.R196Q	13.98	1×	1		82.24	−1.35549764
07-04	421	c.395A>G	p.K132R	38.55	2×	1		69	0.46452687
07-04	439	c.613T>G	p.Y205D	9.96	1×	1	0	94.27	0.41838153
07-04	462	c.583A>T	p.I195F	84.75	1×	1	0	55.72	0.23930621
07-04	475	c.659A>G	p.Y220C	37.62	5×	1	0	72.52	0.21906403
07-04	578	c.524G>A	p.R175H	71.64	7×	1	0	78.5	−0.14494686
07-04	583	c.818G>A	p.R273H	8.79	3×	1	0	66.12	0.25030959
07-04	654	c.524G>A	p.R175H	13.41	7×	1	0	78.5	−0.14494686
07-04	690	c.437G>A	p.W146*	17.31	2×	0	1		0.34844348
07-04	705	c.524G>A	p.R175H	22.86	7×	1		78.5	−0.14494686
07-04	705	c.673-1G>A	NA	20.55	1×	0	1		
07-04	743	c.493C>T	p.Q165*	36.62	2×	0	1		−0.35599844
07-04	873	c.524G>A	p.R175H	76.99	7×	1	0	78.5	−0.14494686
07-04	890	c.527G>T	p.C176F	69.66	1×	1	1	96.33	0.19435669
07-04	909	c.667C>T	p.P223S	4.66	1×	1	0	70.78	-2.66136766
07-04	911	c.832C>T	p.P278S	26.96	1×	1	0	84.34	0.16959628
07-04	913	c.761T>A	p.I254N	5.15	1×	1	0	88.5	−0.11720409
07-04	944	c.517G>T	p.V173L	88	1×	1	0	82.64	0.08713433
07-04	954	c.742C>T	p.R248W	47.22	2×	1	1	84.11	0.07397455
07-04	975	c.524G>A	p.R175H	28.92	7×	1	0	78.5	−0.14494686
07-04	1015	c.743G>A	p.R248Q	83.79	7×	1	1	78.95	−0.03958725
07-04	1056	c.722C>T	p.S241F	7.69	4×	1	1	90.38	−0.0294578
07-04	1096	c.817C>T	p.R273C	89.47	4×	1	0	84.5	−0.12541853
07-04	1100	c.734G>A	p.G245D	22.48	2×	1	1	89.5	−0.00312039
07-04	1107	c.1154delT	p.F385fs*37	52.38	1×	0	1		
07-04	1120	c.388C>G	p.L130V	12.24	2×	1	0	77.51	−0.25882638
07-04	1135	c.659A>G	p.Y220C	33.15	5×	1	0	72.52	0.21906403
07-04	1156	c.710T>A	p.M237K	94.53	1×	1	1	85.52	0.29707147
07-04	1169	c.645T>G	p.S215R	11.03	2×	1	0	89.07	0.00332001
07-04	1171	c.743G>A	p.R248Q	77.72	7×	1	1	78.95	-0.03958725
07-04	1216	c.584T>A	p.I195N	49.47	1×	1	1	87.79	0.11355885
98A	28	c.400T>A	p.F134I	54.37	1×	1	0	56.1	0.18555266
98A	69	c.701A>G	p.Y234C	67.95	1×	1	0	62.9	0.23555926
98A	152	c.1022_1023insT	p.R342fs*5	25.22	1×	0	1		
98A	213	c.743G>A	p.R248Q	94.06	7×	1	1	78.95	−0.03958725
98A	233	c.760A>G	p.I254V	53.28	1×	1	0	34.31	−2.12561332
98A	266	c.490A>G	p.K164E	58.82	2×	1	1	74.74	0.45663438
98A	296	c.419C>A	p.T140N	44.19	1×	1	0	68	−3.19750232
98A	320	c.31G>A	p.E11K	52.61	1×	1	0	10.43	
98A	409	c.814G>A	p.V272M	14.85	2×	1	0	63.4	0.22735985
98A	434	c.823T>C	p.C275R	33.85	1×	1	0	97.76	0.32999589
98A	477	c.818G>A	p.R273H	37.85	3×	1	0	66.12	0.25030959
98A	498	c.722C>T	p.S241F	7.2	4×	1	1	90.38	−0.0294578
98A	536	c.488A>G	p.Y163C	96.77	2×	1	0	70	0.12544937
98A	551	c.800G>C	p.R267P	25.48	1×	1	0	94.88	0.59067636
98A	554	c.722C>T	p.S241F	84.35	4×	1	1	90.38	−0.0294578
98A	568	c.794T>C	p.L265P	90.91	1×	1	0	84	−0.31706353
98A	598	c.814G>A	p.V272M	34.38	2×	1	0	63.4	0.22735985
98A	624	c.711G>A	p.M237I	73.65	3×	1	0	63.68	0.19340041
98A	630	c.159G>A	p.W53*	96.97	1×	0	1		
98A	661	c.439delG	p.V147fs*	71.73	1×	0	1		
98A	691	c.848G>A	p.R283H	48	1×	1	0	48.44	−1.7891287
98A	692	c.722C>T	p.S241F	72.28	4×	1	1	90.38	−0.0294578
98A	695	c.722C>A	p.S241Y	69.83	1×	1	0	89.87	−0.60425762
98A	739	c.376-1G>A	NA	89.19	1×	0	1		
98A	799	c.742C>T	p.R248W	64.37	2×	1	1	84.11	0.07397455
98A	839	c.711G>A	p.M237I	79.38	3×	1	0	63.68	0.19340041
98A	840	c.493C>T	p.Q165*	91.49	2×	0	1		−0.35599844
98A	870	c.608T>A	p.V203E	31.78	1×	1	0	71.79	−0.45171065
98A	919	c.160T>C	p.F54L	16.94	1×	1	0	2.55	
98A	941	c.376-2A>G	NA	78.13	1×	0	1		
98A	1076	c.395A>G	p.K132R	76.67	2×	1	0	69	0.46452687
98B	35	c.725G>C	p.C242S	28.98	1×	1	0	86.74	0.26799062
98B	49	c.637C>T	p.R213*	87.21	1×	0	1		
98B	69	c.25A>G	p.S9G	15.71	1×	1	0	16.9	
98B	71	c.154C>T	p.Q52*	84.08	1×	0	1		
98B	87	c.577C>A	p.H193N	69.93	1×	1	1	73.11	0.17590301
98B	98	c.711G>A	p.M237I	15.51	3×	1		63.68	0.19340041
98B	98	c.490A>G	p.K164E	16.23	2×	1	1	74.74	0.45663438
98B	103	c.993+1G>A	NA	89.86	1×	0	1		
98B	126	c.785G>T	p.G262V	43.67	1×	1		88.2	0.28619807
98B	126	c.553_559+ 2delAGCGATGgt	p.?	47.57	1×	0	1		
98B	199	c.645T>G	p.S215R	26.99	2×	1		89.07	0.00332001
98B	199	c.560-1G>A	NA	19.44	1×	0	1		
98B	201	c.472delC	p.R158fs*	16.66	1×	0			
98B	201	c.320_321insA	p.Y107fs*1	18.96	1×	0	1		
98B	220	c.715A>G	p.N239D	57.03	1×	1	1	49.44	−0.02472517
98B	226	c.818G>A	p.R273H	81.2	3×	1	0	66.12	0.25030959
98B	275	c.329G>T	p.R110L	34.88	1×	1	0	28.14	
98B	292	c.659A>G	p.Y220C	64.2	5×	1	0	72.52	0.21906403
98B	332	c.388C>G	p.L130V	58.54	2×	1	0	77.51	−0.25882638
98B	352	c.659A>G	p.Y220C	72.05	5×	1		72.52	0.21906403
98B	352	c.438G>A	p.W146*	7.69	2×	0	1		0.34844348
98B	358	c.817C>T	p.R273C	76	4×	1	0	84.5	−0.12541853
98B	408	c.743G>A	p.R248Q	77.17	7×	1	1	78.95	−0.03958725
98B	458	c.734G>A	p.G245D	54.1	2×	1	1	89.5	−0.00312039

**Table 2:** Median overall survival rates of AMLSG patients with and without *TP53*-mutated acute myeloid leukemia (AML) and with respect to *TP53*-specific functional scoring systems.

Variable	Group	Median	95% CI
TP53	mut	6.5	5.0–8.2
	wt	33.6	28.4–45.0
Disruptive	no	7.3	5.0–11.7
	yes	5.5	3.7–7.9
Missense	no	5.1	2.9–14.0
	yes	6.6	5.0–8.9
EAp53	<75	8.2	5.4–12.9
	≥75	5.5	4.6–8.7
RFS	≤−1	13.4	12.9–NA
	>−1	6.3	5.0–8.2
RFS AML	≤−0.135	12.9	6.9–24.7
	>−0.135	5.5	3.8–8.2

Abbreviations: CI, confidence interval; mut, *TP53*-mutated; wt, wild type *TP53*; Disruptive, disruptive mutations; Missense, missense mutations; EAp53, Evolutionary Action p53 Score; RFS, Relative Fitness Score; RFS AML, AML-specific RFS; NA, not applicable—median survival not reached.

**Table 3:** Multivariable Cox proportional hazards regression analysis of the effect of different functional mutation scoring systems on overall survival (OS) and event-free survival (EFS) in patients with *TP53*-mutated acute myeloid leukemia (AML).

		OS		EFS	
Variable	Category	HR (95% CI)	*p*	HR (95% CI)	*p*
Missense	no	1		1	
	yes	1.0 (0.49–2.05)	0.998	1.49 (0.73–3.06)	0.277
Disruptive	no	1		1	
	yes	1.68 (0.99–2.86)	0.056	1.18 (0.71–1.96)	0.533
EAp53	<75	1		1	
	≥75	1.12 (0.65–1.93)	0.678	1.41 (0.82–2.4)	0.212
RFS	≤−1	1		1	
	>−1	4.26 (0.91–19.88)	0.065	4.08 (0.88–18.94)	0.072
RFS AML	≤−0.135	1		1	
	>−0.135	2.14 (1.15–4.0)	0.017	1.97 (1.06–3.69)	0.033

The models are adjusted for age, gender, white blood cell count, cytogenetic risk, type of AML, and *TP53* variant allele frequency. Abbreviations: HR, hazard ratio; CI, confidence interval; EAp53, Evolutionary Action p53 Score; RFS, Relative Fitness Score; RFS AML, AML-specific RFS.

**Table 4:** Classification of amino acids by polarity and charge.

Nonpolar	Polar, Negatively Charged	Polar, No Charge	Polar, Positively Charged
Phenylalanine F	Aspartic acid D	Cysteine C	Histidine H
Methionine M	Glutamic acid E	Asparagine N	Lysine K
Tryptophan W		Glutamine Q	Arginine R
Isoleucine I		Threonine T	
Valine V		Tyrosine Y	
Leucine L		Serine S	
Alanine A		Glycine G	
Proline P			

## Supplementary Materials

The following are available online at https://www.mdpi.com/2072-6694/12/3/637/s1. Table S1: Median event-free survival rates of AMLSG patients with and without TP53-mutated AML and with respect to TP53 functional scoring systems; Table S2: Cox proportional hazards regression analysis of the effect of different functional mutation scoring systems and patient characteristics on overall survival—univariate analysis; Table S3: Cox proportional hazards regression analysis of the effect of different functional mutation scoring systems and patient characteristics on event-free survival—univariate analysis; Table S4: Sensitivity and specificity values of the various thresholds for the EAp53 score and the “Relative Fitness Score” (RFS); Figure S1: Event-free survival probabilities for patients with *TP53*-mutated AML according to functional mutation scoring systems; Figure S2: Overall and event-free survival probabilities for patients with *TP53*-mutated AML according to the RFS with a cut-off set at −1; Figure S3: Receiver operating characteristics analysis for the “Relative Fitness Score” as a predictor for 1-year mortality; Figure S4: Boxplots of the “EAp53 score” and the “Relative Fitness Score” for patients with *TP53*-mutated AML according to the survival status at 1 year.
