# Supplementary material for: Functional Classification of TP53 Mutations in Acute Myeloid Leukemia
Source: Cancers (Basel). 2020 Mar 10;12(3):637. doi: 10.3390/cancers12030637 (PMC7139772; doi:10.3390/cancers12030637)
Supplement: Supplementary file 1 [file cancers-12-00637-s001.pdf]

cancers

# Functional Classification of *TP53* Mutations in Acute Myeloid Leukemia

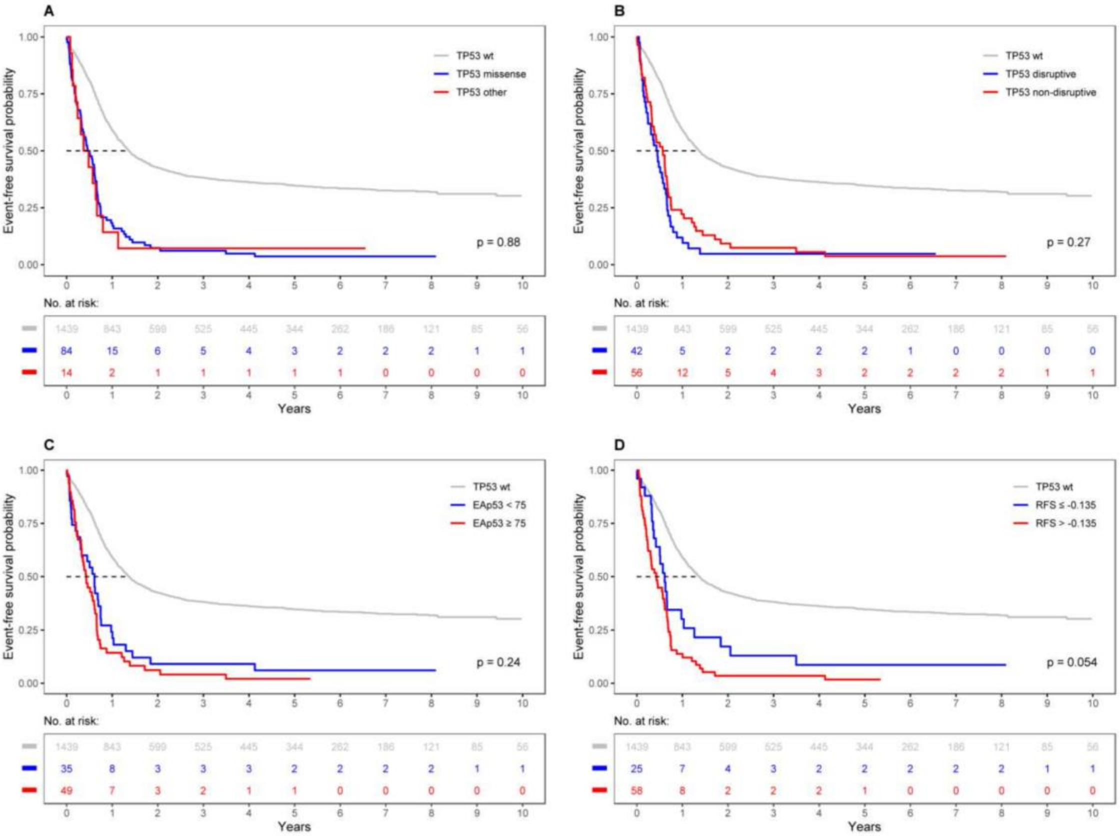


**Figure S1.** Event-free survival probabilities for patients with *TP53* mutated AML according to functional mutation scoring systems. (**A**) comparison of missense *TP53* mutations versus other types (nonsense and splice-site mutations, insertions and deletions). (**B**) comparison of disruptive versus non-disruptive mutations. (**C**) classification according to the "Evolutionary Action p53 Score" (EAp53). (**D**) classification based on the "Relative Fitness Score" (RFS) with the AML-specific threshold of -0.135. All P-values refer to the comparison of *TP53* mutated groups, survival of *TP53* wild-type patients is shown as a reference.

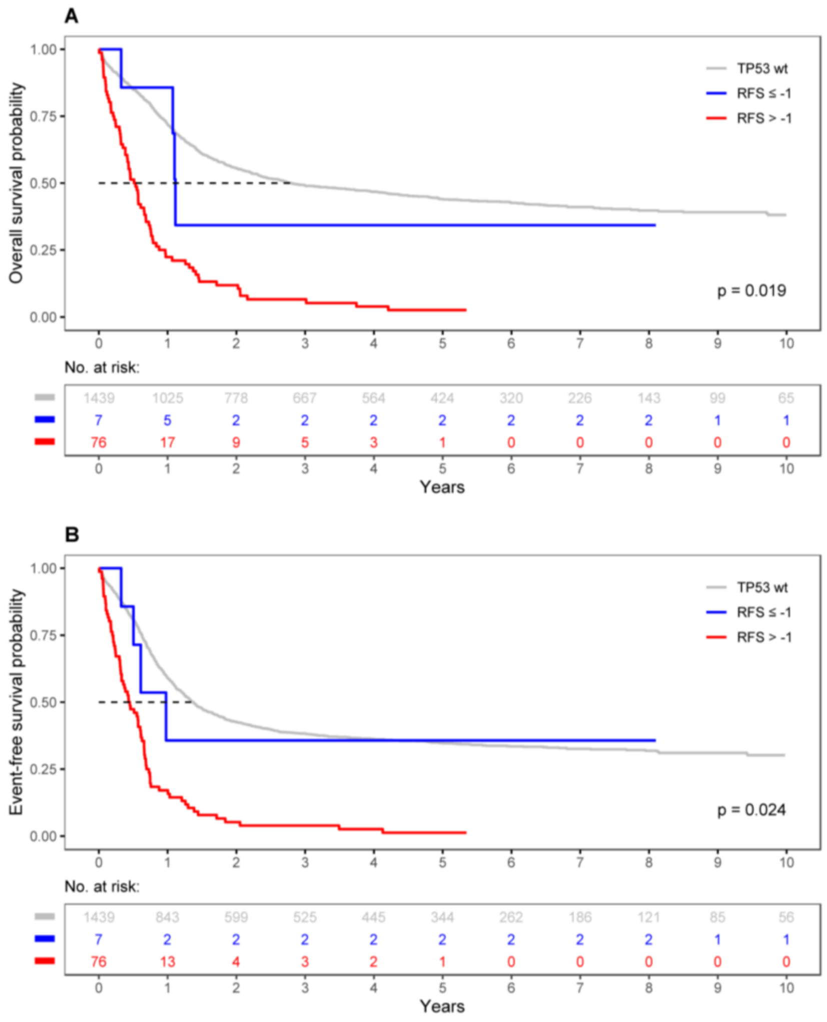


**Figure S2.** Overall and event-free survival probabilities for patients with *TP53* mutated AML according to the RFS with a cut-off set at -1.

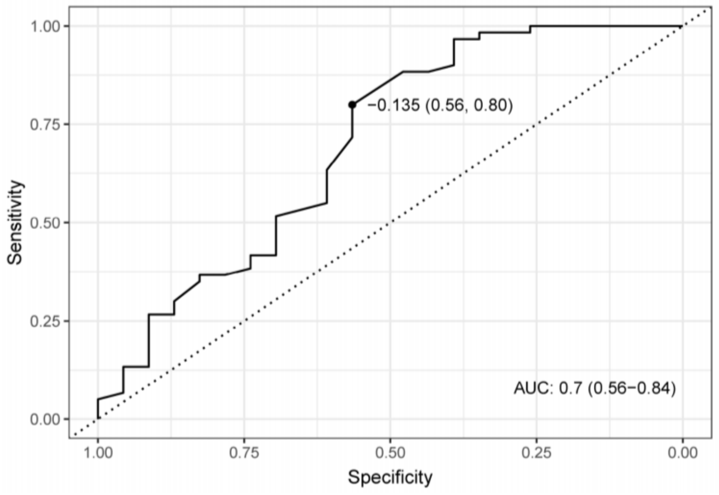


**Figure S3.** Receiver operating characteristics analysis for the "Relative Fitness Score" as a predictor for 1-year mortality. Abbreviation: AUC, area under the curve.

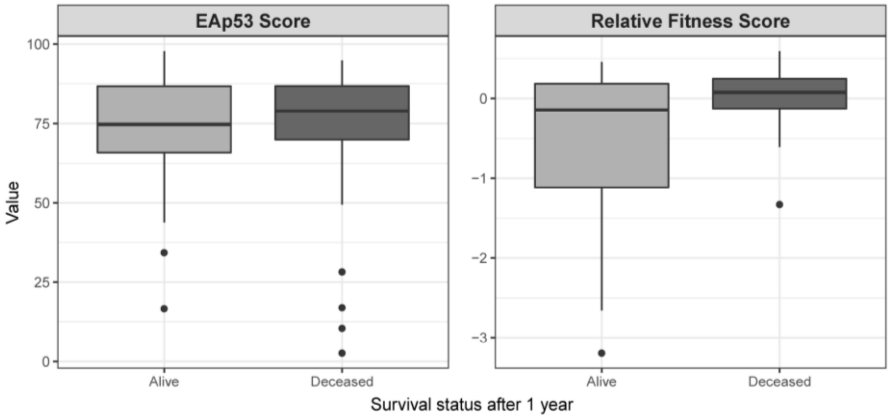


**Figure S4.** Box plots of the "EAp53 Score" and the "Relative Fitness Score" for patients with *TP53* mutated AML according to the survival status at 1 year.

**Table S1.** Median event-free survival rates of AMLSG patients with and without *TP53* mutated AML and with respect to *TP53* functional scoring systems. Abbreviations: mut, *TP53* mutated; wt, *TP53* wild-type; CI, confidence interval; EAp53, "Evolutionary Action p53 Score"; RFS, "Relative Fitness Score"; RFS AML, AML-specific RFS; NA, not applicable—median survival not reached.

| Variable | Group | Median | 95% CI |
|---|---|---|---|
| *TP53* | mut | 5.7 | 4.3–7.4 |
| | wt | 16.5 | 15.0–18.2 |
| **Disruptive** | no | 6.8 | 4.0–8.0 |
| | yes | 5.3 | 3.0–7.7 |
| **Missense** | no | 5.1 | 2.9–13.6 |
| | yes | 5.8 | 4.0–7.5 |
| **EAp53** | < 75 | 7.3 | 3.7–9.1 |
| | ≥ 75 | 5.2 | 4.0–7.5 |
| **RFS** | ≤ −1 | 11.7 | 6.0–NA |
| | > −1 | 5.3 | 4.0–7.4 |
| **RFS AML** | ≤ −0.135 | 7.3 | 5.0–15.1 |
| | > −0.135 | 5.2 | 3.6–7.4 |

**Table S2.** Cox proportional hazards regression analysis of the different functional mutation scoring systems and patient characteristics on overall survival–univariable analysis. The *TP53* variant allele frequency (VAF) is assessed as a continuous and a binary variable. Abbreviations: HR, hazard ratio; CI, confidence interval; EAp53, "Evolutionary Action p53 Score"; RFS, "Relative Fitness Score"; RFS AML, AML-specific RFS.

| Variable | Category | HR (95% CI) | *p* |
|---|---|---|---|
| **Disruptive** | no | 1 (Ref.) | 0.064 |
| | yes | 1.49 (0.98–2.26) | |
| **Missense** | no | 1 (Ref.) | 0.492 |
| | yes | 0.81 (0.45–1.47) | |
| **EAp53 score** | <75 | 1 (Ref.) | 0.488 |
| | ≥75 | 1.17 (0.75–1.84) | |
| **RFS** | ≤−1 | 1 (Ref.) | 0.026 |
| | >−1 | 3.15 (1.15–8.67) | |
| **RFS AML** | ≤−0.135 | 1 (Ref.) | 0.018 |
| | >−0.135 | 1.83 (1.11–3.02) | |
| **Age** | | 1.05 (1.02–1.07) | <0.001 |
| **Gender** | male | 1 (Ref.) | 0.460 |
| | female | 1.17 (0.77–1.78) | |
| **White blood cell count** | | 1.01 (1.00–1.02) | 0.045 |
| **Cytogenetic risk** | high | 1 (Ref.) | 0.161 |
| | intermediate | 0.47 (0.21–1.02) | |
| | low | 0.83 (0.20–3.41) | |
| **Type of AML** | AML | 1 (Ref.) | 0.193 |
| | secondary AML | 1.47 (0.53–4.04) | |
| | Therapy-related AML | 0.58 (0.30–1.13) | |
| **VAF** | | 1.00 (1.00–1.01) | 0.428 |
| **VAF** | <20 | 1 (Ref.) | 0.398 |
| | ≥20 | 0.79 (0.46–1.36) | |

**Table S3.** Cox proportional hazards regression analysis of the different functional mutation scoring systems and patient characteristics on event-free survival–univariable analysis. The *TP53* variant allele frequency (VAF) is assessed as a continuous and a binary variable. Abbreviations: HR, hazard ratio; CI, confidence interval; EAp53, "Evolutionary Action p53 Score"; RFS, "Relative Fitness Score"; RFS AML, AML-specific RFS.

| Variable | Category | HR | *p* |
|---|---|---|---|
| **Disruptive** | no | 1 (Ref.) | 0.274 |
| | yes | 1.26 (0.83–1.91) | |
| **Missense** | no | 1 (Ref.) | 0.882 |
| | yes | 0.96 (0.53–1.72) | |
| **EAp53 score** | <75 | 1 (Ref.) | 0.240 |
| | ≥75 | 1.31 (0.83–2.06) | |
| **RFS** | ≤−1 | 1 (Ref.) | 0.032 |
| | >−1 | 3.05 (1.10–8.43) | |
| **RFS AML** | ≤−0.135 | 1 (Ref.) | 0.056 |
| | >−0. 135 | 1.63 (0.99–2.68) | |
| **Age** | | 1.05 (1.03–1.08) | <0.001 |
| **Gender** | male | 1 (Ref.) | 0.715 |
| | female | 1.08 (0.71–1.64) | |
| **White blood cell count** | | 1.01 (1.00–1.02) | 0.004 |
| **Cytogenetic risk** | high | 1 (Ref.) | 0.122 |
| | intermediate | 0.44 (0.20–0.97) | |
| | low | 0.74 (0.18–3.06) | |
| **Type of AML** | AML | 1 (Ref.) | 0.099 |
| | secondary AML | 1.31 (0.48–3.60) | |
| | therapy-related AML | 0.50 (0.26–0.97) | |
| **VAF** | | 1.00 (0.99–1.01) | 0.915 |
| **VAF** | <20 | 1 (Ref.) | 0.357 |
| | ≥20 | 0.78 (0.46–1.33) | |

**Table S4.** Sensitivity and specificity values of the various thresholds for the "EAp53 Score" and the "Relative Fitness Score" (RFS). For each of the investigated thresholds, we stated the number of patients that were below and above the threshold in the groups "alive" and "deceased" after 1 year. The resulting sensitivity and specificity values are presented with their respective 95% confidence intervals (CI).

| Scoring System | Threshold | Below/above (deceased) | Below/above (alive) | Sensitivity (95% CI) | Specificity (95% CI) |
|---|---|---|---|---|---|
| **EAp53** | **75** | 23/38 | 12/11 | 62.3 (49.0–74.4) | 52.2 (30.6–73.2) |
| **RFS** | **−1** | 1/59 | 6/17 | 98.3 (91.1–100) | 26.1 (10.2–48.4) |
| **RFS AML** | **−0.135** | 12/48 | 13/10 | 80.0 (70.0–90.0) | 56.5 (34.8–73.9) |

**Investigators and centers of the German-Austrian AML Study Group.**

| | |
|---|---|
| Karin T. Mayer M.D. | Universitätsklinikum Bonn, Bonn Germany |
| Bernd Hertenstein, M.D. | Klinikum Bremen Mitte, Bremen, Germany |
| Thomas Schroeder, M.D. | Universitätsklinikum Düsseldorf, Düsseldorf, Germany |
| Mohammed Wattad, M.D. | Kliniken Essen Süd, Ev. Krankenhaus Essen Werden GmbH, Essen, Germany |
| Swen Wessendorf, M.D. | Klinikum Esslingen, Esslingen, Germany |
| Hans Gunter Derigs, M.D. | Klinikum Frankfurt Höchst GmbH, Frankfurt, Germany |
| Michael Lübbert, M.D. | Universitätsklinikum Freiburg, Freiburg, Germany |
| Alexander Burchardt, M.D. | Universitätsklinikum Giessen, Giessen, Germany |
| Volker Runde, M.D. | Wilhelm Anton Hospital, Goch, Germany |
| Gerald Wulf M.D. | Universitätsklinikum Göttingen, Göttingen, Germany |
| Walter Fiedler, M.D. | Universitätsklinikum Hamburg Eppendorf, Hamburg, Germany |
| Hans Salwender, M.D. | Asklepios Klinik Altona, Hamburg, Germany |
| Elisabeth Lange, M.D. | Evangelisches Krankenhaus Hamm, Hamm, Germany |
| Andrea Sendler, M.D. | Klinikum Hanau, Hanau, Germany |
| Arnold Ganser, M.D. | |
| Michael Heuser, M.D. | Medizinische Hochschule Hannover, Hannover, Germany |
| Felicitas Thol M.D. | |
| Hartmut Kirchner, M.D. | KRH Klinikum Siloah, Hannover, Germany |
| Uwe Martens, M.D. | SLK Kliniken GmbH Heilbronn, Heilbronn, Germany |
| Jörg-Thomas Bittenbring, M.D. | Universitätsklinikum des Saarlandes, Homburg, Germany |
| David Nachbaur, M.D. | Universitätsklinikum Innsbruck, Innsbruck, Austria |
| Mark Ringhoffer, M.D. | Städtisches Klinikum Karlsruhe GmbH, Karlsruhe, Germany |
| Heinz A. Horst, M.D. | Universitätsklinikum Schleswig Holstein–Campus Kiel, Kiel, Germany |
| Stephan Kremers, M.D. | Caritas Krankenhaus Lebach, Lebach, Germany |
| Andreas Petzer, M.D. | Krankenhaus der Barmherzigen Schwestern Linz, Linz, Austria |
| Gerhard Heil, M.D. | Klinikum Ludenscheid, Ludenscheid, Germany |
| Thomas Kindler, M.D. | Universitätsklinikum Mainz, Mainz, Germany |
| Katharina Götze, M.D. | Klinikum rechts der Isar der Technischen Universität München, München, Germany |
| Sabine Struve, M.D. | Klinikum Schwabing, München, Germany |
| Peter Schmidt, M.D. | Städtisches Klinikum Neunkirchen, Neunkirchen, Germany |
| Ali Nuri Hünerliturkoglu, M.D. | Lukaskrankenhaus GmbH Neuss, Neuss; Germany |
| Claus Henning Köhne, M.D. | Klinikum Oldenburg, Oldenburg, Germany |
| Gregg Frost, M.D. | Caritas Klinik St. Theresia, Saarbrücken, Germany |
| Richard Greil, M.D. | Universitätsklinikum der Paracelsus Medizinischen Universität Salzburg, Salzburg, Austria |

| | |
|---|---|
| Jochen Greiner, M.D. | Diakonie Klinikum Stuttgart, Stuttgart, Germany |
| Heinz Kirchen, M.D. | Krankenhaus der Barmherzigen Brüder, Trier, Germany |
| Hans Gernot Biedermann, M.D. | Kreisklinik Trostberg, Trostberg, Germany |
| Helmut Salih, M.D. | Universitätsklinikum Tübingen, Tübingen, Germany |
| Hartmut Döhner, M.D. | Ulm, Ulm, Germany |
| Paul Graf La Rosée, M.D. | Schwarzwald Baar Klinikum Villingen Schwenningen GmbH, Villingen Schwenningen, Germany |
| Elisabeth Koller, M.D. | Hanuschkrankenhaus, Wien, Austria |
| Aruna Raghavachar, M.D., | Helios Klinikum Wuppertal, Wuppertal, Germany |
